# Current overview of the mechanistic pathways and influence of physicochemical parameters on the microbial synthesis and applications of metallic nanoparticles

**DOI:** 10.1007/s00449-025-03190-w

**Published:** 2025-06-25

**Authors:** Sharad Bhatnagar, Hideki Aoyagi

**Affiliations:** 1 Institute of Life and Environmental Sciences, University of Tsukuba, 1-1-1, Tennodai, Tsukuba, Ibaraki 305-8572 Japan; 2 Microbiology Research Center for Sustainability (MiCS), University of Tsukuba, 1-1-1, Tennodai, Tsukuba, Ibaraki 305-8572 Japan; 3 Tsukuba Institute for Advanced Research (TIAR), University of Tsukuba, 1-1-1, Tennodai, Tsukuba, Ibaraki 305-8577 Japan

**Keywords:** Nanoparticles, Physicochemical factors, Microorganism-mediated synthesis, Biosynthesis, Mechanistic overview

## Abstract

Microbe-assisted synthesis of metallic nanoparticles (NPs) has carved a niche among different NP generation methods owing to its simplicity, non-toxicity, low energy requirements, and potential scalability. Microorganisms have ability to produce NPs both intracellularly and extracellularly due to the presence of enzymes, proteins, and other biomolecules that can act as reducing and capping agents. However, a complete mechanistic understanding of this biosynthesis remains elusive. Biosynthesis is influenced by a myriad of factors, such as pH, temperature, reactant concentrations, reaction time, and light. The physicochemical factors associated with the synthesis process affect the morphological, biological, and catalytic properties of the NPs produced. This review focuses on the current paradigm and gaps in our understanding of microbial production pathways and the effects of physicochemical factors on the synthesis and application of various types of metallic NPs. The surveyed literature clearly elucidated the effect of these factors on the size, shape, dispersity, surface properties, and the reaction kinetics. The variations in morphological and surface properties were found to affect the performance of NPs in different applications such as catalysis, antimicrobial, and anticancer activities. Understanding the mechanistic pathways and the influence of physicochemical factors on synthesis can be potentially beneficial for the production of NPs with controlled shapes and sizes, tailored for specific applications.

## Introduction

Nanoparticles (NPs) are particles that have a diameter of 1–100 nm and exhibit properties completely different from those of their constituent bulk material owing to their size. At their size, quantum effects dominate, imparting unique properties to these particles. This has made NPs useful for a wide variety of applications in different fields such as bioremediation, for heavy metal removal or dye degradation [1]; biomedical fields, as antimicrobial, antibiofilm, anticancer agents, for bioimaging, and drug delivery [2–4]; and in agriculture, as biosensors, pesticides, fertilizers, and food packaging, among many others [5].

NP production can be divided into a top-down approach, in which the bulk material is reduced to the nanoscale, and a bottom-up approach, in which the particles are built up from the atomic and molecular scales. These methods can be further subdivided into physical, chemical, and biological processes. Physical processes include thermal vapor evaporation, laser ablation, sputtering, etc., whereas chemical processes comprise sol–gel and polyol processes, as well as chemical reductions, among others [6]. The major drawbacks associated with these techniques are the requirement of a large amount of energy or the production of chemical wastes that are harmful to the environment. In contrast, plants and microorganisms can generate NPs in a simple and ecofriendly manner [7]. Moreover, the use of microorganisms provides numerous advantages, such as cost and energy efficiency, scalability, biocompatibility, and versatility. The reduction and capping facilitated by various biological agents make these particles valuable for biomedical applications. Although plant-mediated synthesis of NPs has been shown to be important, NPs synthesized via these methods might display some polydispersity due to the phytochemicals used and can lead to differences in yields due to seasonal variations [4]. In contrast, microorganisms can be used as factories for the cost-effective synthesis of NPs by reducing or avoiding environmentally toxic chemicals and can easily be scaled up.

Many different types of microorganisms such as algae, bacteria, actinomycetes, fungi, and yeasts have been used for the synthesis of NPs. When considering microorganisms for synthesis of NPs, the importance of microbial types and species cannot be overstated. For example, intracellular or extracellular synthesis is often affected by the choice of microbial species. Similarly, fungi have become an organism of choice for NP synthesis owing to their yields, high tolerance and intracellular metal intake capacity, and ease of growth on solid substrates [8]. The higher productivity of fungi is generally considered to be a result of their ability to produce a variety of enzymes and more amount of proteins [9]. A list of recent advances in the microbial production of various metallic and non-metallic NPs is provided in Table 1 to provide an idea about versatility of microbial NP synthesis. However, the objective of this review is to understand the various aspects of NP generation and control parameters involved in the process, and therefore, detailed discussion on NPs produced from various microbial sources has not been considered. These details can be found in several recent reviews that have covered production of NPs from various microbial species thoroughly [10, 11]. Even now, the mechanisms of action involved in the production of NPs via microorganisms are not comprehensively understood. Moreover, the effect of physicochemical parameters on NP generation needs to be studied comprehensively to devise efficient microbe-mediated NP generation processes before these processes can be employed at large scale as an alternative to the current synthesis processes. Therefore, this review focuses on the current understanding of mechanistic models of microbe-mediated NP synthesis, examines the current literature in the field, and emphasizes the effects of physicochemical parameters on the synthesis and applications of microbially generated NPs.

**Table 1:** Recent advances in nanoparticle generation by various microorganisms

S. no.	Microorganism	Type	NPs	Properties	Applications	References
1	*Synechococcus moorigangae*	Cyanobacteria	Au	2.5–19.9 nm	Antioxidant, Antibacterial	[12]
2	*Spirulina platensis*	Cyanobacteria	Ag	28.70 ± 5.40 nm (DLS)	Antibacterial	[13]
3	*Chlorella* sp.	Microalgae	TiO_2_	10–20 nm	Photocatalysis	[14]
4	*Nannochloropsis oceanica* CASA CC201	Microalgae	Se	78–396 nm	Antioxidant	[15]
5	*Chlorella vulgaris*	Microalgae	ZnO	35–90 nm	Antibacterial, Antioxidant	[16]
6	*Streptomyces albogriseolus*	Actinomycete	Au	5.42–13.34 nm	Anticancer	[17]
7	*Streptomyces enissocaesilis* BS1	Actinomycete	Ag	32.2 nm (mean size)	Antibacterial, Anticancer	[18]
8	*Stenotrophomonas bentonitica*	Bacteria	Se	50–200 nm	–	[19]
9	*Stenotrophomonas* sp. BS95	Bacteria	CuO	35.24 ± 4.64 nm (crude) 43.68 ± 2.31 nm (calcined)	Antibacterial, Antioxidant, Anticancer	[20]
10	*Planococcus maritimus* MBP-2	Bacteria	Ag	24.9 nm	Antimicrobial, Anticancer	[21]
11	*Burkholderia sp.* EIKU21	Bacteria	ZnO	55.08 ± 2.28 nm	Antibacterial	[22]
12	*Talaromyces haitouensis*	Fungi	Se-BiO-CuO	66–80 nm	Antibacterial	[23]
13	*Botrytis cinerea* *Trichoderma atroviride* *Trichoderma asperellum* *Alternaria* sp. *Ganoderma sessile*	Fungi	Au	92.9 nm 24.7 nm 16.4 nm 9.5 nm 13.6 nm	SERS amplification	[24]
14	*Talaromyces purpureogenus*	Fungi	Au	16–40 nm	Catalytic degradation	[25]
15	*Talaromyces islandicus* VSGF1	Fungi	ZnO	22–34 nm (XRD)	Antimicrobial, Anti-inflammatory, Pesticide	[26]

DLS: Dynamic Light Scattering, XRD: X-ray diffraction

## Mechanistic overview of the microbe-assisted synthesis of nanoparticles

**Fig. 1:**
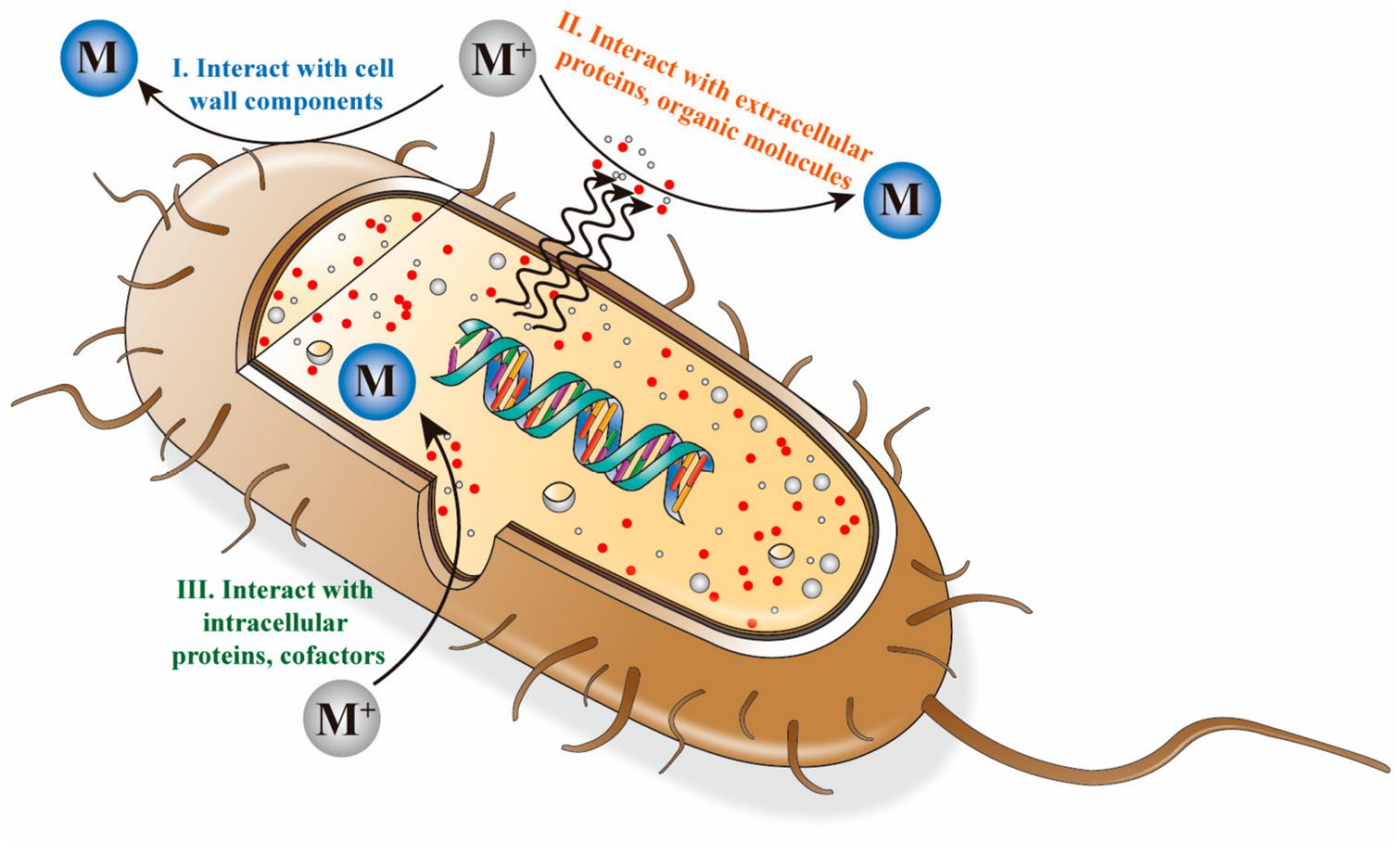
Representation of the intracellular and extracellular nanoparticle production processes. M^+^ and M refers to metal ions and metal nanoparticles, respectively. Adapted from [30], MDPI (CC BY 4.0.)

The potential of microorganisms to generate NPs has been linked to their ability to resist heavy metals by either accumulation or detoxification via molecular mechanisms [27, 28]. The mechanism of NP production by microorganisms might vary from organism to organism; however, in general, this process takes place either intracellularly, where precursor molecules are taken up by the organism, or extracellularly, where they are reduced outside the cell [29, 30]. The production of metallic NPs, such as silver and gold, is thought to be assisted by enzymes and proteins present in or in the vicinity of cells [29] (Fig. 1). These enzymes and proteins act as reducing and capping agents. Initial reports suggested that nitrate-reducing enzymes play a major role in NP generation [27]. This reaction is related to microbial resistance to metal ions. NADH-dependent reductases and shuttle quinones, such as anthraquinones, naphthoquinones, and hydroquinones, are thought to play important roles in this process [31]. Different mechanisms underlying NP biogeneration are discussed in the following sections.

### Enzyme-based mechanisms

The initial mechanism of silver nanocrystal catalysis by *Bacillus licheniformis* revealed the role of NADH-dependent reductase as a carrier and electrons from NADH as a reducing agent. The production of nitrate reductase is also affected by nitrate ions, and a probable mechanistic pathway involved is enzymatic reduction via the electron shuttle [32, 33]. This mechanism was further elucidated by Vaidyanathan et al. [34] who used various inhibitors of nitrate reductase to demonstrate that a decrease in yield occurs with an increase in inhibition. Moreover, nitrate reductase activity was optimized to enhance productivity of the method. Recently, computational profiling of nitrate reductase was used to understand the physicochemical characteristics and structural orientation of this enzyme to develop a more efficient NP production system [35]. A similar mechanism of synthesis for silver NPs (AgNPs) was demonstrated by the actinobacteria, *Streptomyces* sp. LK3, where a process mediated by nitrate reductase was proposed, based on results of a nitrate reductase test. This organism was found to reduce not only nitrate to nitrite but also nitrite to nitrogenous gases [36].

**Fig. 2:**
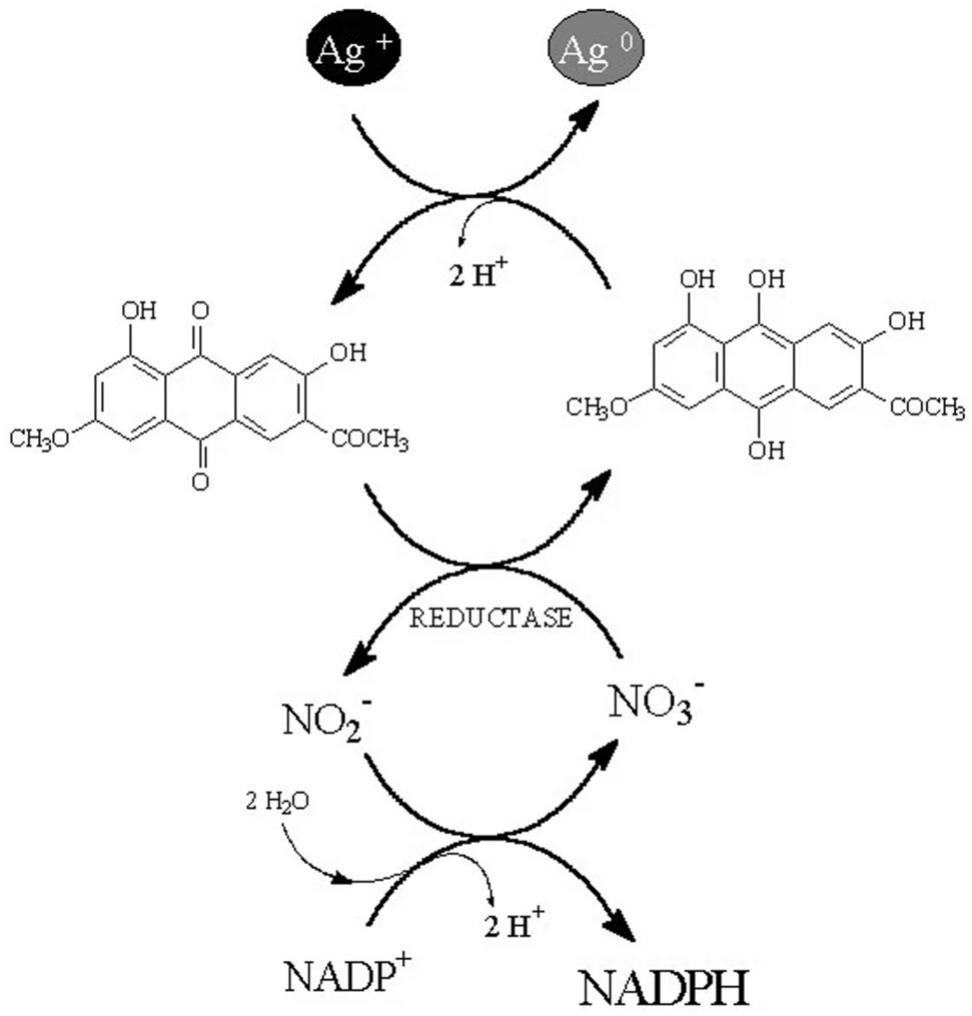
Nitrate reductase-mediated pathway for the biosynthesis of silver nanoparticles. Adapted from [31], BioMed Central Ltd. (CC BY 2.0.)

Fungi have also been shown to reduce bulk metals to NPs via nitrate reductase. The cell-free filtrates of *Fusarium oxysporum* were used to synthesize AgNPs, which were linked to the presence of nitrate reductase. This study also showed that productivity, monodispersity, and size distribution are related to reductase enzyme activity [37]. However, Durán et al. [31] had earlier shown that not only reductases but also anthraquinones are necessary to participate in the electron shuttle for the reduction process. Another research showed that *F. oxysporum* had the ability to reduce Ag^+^ ions extracellularly, whereas no such capability was observed in *F. moniliforme* (currently *F. verticillioides*), even though both contained intra- and extracellular reductase enzymes [38]. Therefore, the difference observed in anthraquinone production was considered the major factor affecting NP production (Fig. 2). This mechanism showed that α-NADPH-dependent nitrate reductase, along with phytochelatin as a capping agent, was the possible mechanism involved in the synthesis of AgNPs from *F. oxysporum*. Nitrate reductase optimization via response surface methodology (RSM) was recently demonstrated for *Aspergillus terreus* N4, with the enzyme located intracellularly and in the periplasmic space. The enzyme fractions were able to produce AgNPs in less than 20 min, with a size range of 25–30 nm [39]. A similar mechanism has previously been described for gold NP (AuNP) production, where the protein extract from *F. oxysporum* was concentrated, and cofactors removed through dialysis. This concentrate could not synthesize AuNPs without the external addition of stoichiometric amounts of NADH. Hence, the authors concluded that the reduction of AuCl_4_^−^ ions was mediated by NADH-dependent reductases present in the extract [40]. Similarly, Nangia et al. [41] demonstrated the intracellular synthesis of AuNPs via *Stenotrophomonas maltophilia* and suggested that the possible mechanism of synthesis involves the reduction of AuCl_4_^−^ ions to Au^+^, followed by reduction to Au^0^ in the presence of an NADPH-dependent reductase enzyme. Although the current understanding of NP synthesis shows that NADPH-dependent nitrate reductase is central to the mechanism involved, a recent report showed that under in vitro conditions, using NADPH alone is sufficient to reduce silver nitrate (AgNO_3_) to AgNPs. Moreover, the addition of nitrate reductase slowed the reaction and increased the particle size distribution [42]. These data indicate that gaps still exist in our mechanistic understanding of the Ag^+^ reduction process.

The synthesis mechanism of other types of NPs via enzymatic reduction have also been previously reported. An early report on *F. oxysporum* implied that sulfate reductase enzymes are responsible for the reduction of CdSO_4_ to extracellularly produce cadmium sulfide NPs (CdSNPs) [43]. Sulfate-reducing bacteria were employed by Watson et al. [44] to synthesize iron sulfide nanomaterials. *Desulfovibrio desulfuricans*, a sulfate-reducing bacterium, has been used to synthesize palladium nanomaterials via a basic formate hydrogenlyase complex. It was assumed that the crystal nuclei formed by the target ions interacted with the localized surface-binding sites and were possibly reduced by hydrogenase activity. The growth of the crystal structure was dependent on the cell surface architecture [45]. A similar mechanism was used to explain the production of intracellular palladium NPs (PdNPs) by *Shewanella oneidensis* MR-1 cells [46]. Recently, *Shewanella* sp. CNZ-1 was used to produce Pd(0)NPs from the reduction of Pd(II), and the authors asserted that this organism was more efficient at generating PdNPs than *S. oneidensis* MR-1. The reduction was mediated by hydrogenases and some amides [47]. Deplanche et al. [48] demonstrated the importance of periplasmic hydrogenases in the bioreduction of Pd(II) to Pd(0) by *Escherichia coli*. The [NiFe] hydrogenases involved in the reaction were membrane bound, and the location of Pd(0) deposits in the cell was dependent on the subcellular localization of the hydrogenases. In another study, *Shewanella algae* was used in conjunction with hydrogen as an electron donor under anaerobic conditions to generate AuNPs intracellularly. The mechanism of synthesis is thought to be mediated by the hydrogenase enzyme catalyzing reduction of AuCl_4_^−^ ions using hydrogen as the electron donor [49].

In addition to these enzymes, studies have indicated that cysteine desulfhydrase (C-S lyase) is a key contributor to the synthesis of metal sulfide NPs as it can control crystal growth by producing S^2−^ ions by acting on cysteine-rich proteins. The photosynthetic bacterium, *Rhodopseudomonas palustris*, was used to synthesize CdSNPs via the intracellularly localized C-S lyase. The authors showed that CdSNPs were synthesized intracellularly and then released from the cells [50]. Similarly, *Rhodobacter sphaeroides* was used to synthesize lead sulfide NPs (PbSNPs), and this synthesis was attributed to the presence of C-S lyase. Moreover, the authors showed that the culture time affected C-S lyase activity, which in turn affected the rate of reaction between Pb and S^2−^, resulting in changes in the size of the formed PbSNPs [51]. Other enzymes, such as xylanases and laccases, have also been used to produce NPs [52, 53]. These studies show that for extracellular synthesis processes, the key enzymes and cofactors involved are present outside the cell or on the cell surface, whereas the intracellular processes are clearly dependent on the localization of enzymes. The metal ions absorbed by the cells are carried to these sites for reduction, followed by accumulation inside the cell, and sometimes followed by expulsion of the nanomaterial from the cell. The studies on the enzymatic synthesis show that nitrate reductases are among the most commonly used enzymatic route for NP generation, but even after multiple studies some gaps still remain in the understanding of the synthesis mechanism. However, the ability of microbes to utilize various enzymes for the production of different NPs demonstrates the flexibility offered by the microorganism-mediated synthesis route.

### Protein/amino acid-based mechanisms

In addition to enzymes, proteins, peptides, and amino acids are important for the reduction and capping of NPs. The interaction of silver-binding peptides with silver metal nuclei in aqueous AgNO_3_ solution, and the consequent reduction that occurs when the peptides interact with metal clusters, has been demonstrated previously [54]. Selvakannan et al. [55] synthesized AgNPs from silver sulfate using tyrosine as a reducing agent under alkaline conditions. This reducing ability of tyrosine was attributed to ionization of the phenolic group under alkaline conditions, which enabled the electron transfer of phenolate ions to Ag^+^, leading to the formation of AgNPs and their conversion into semiquinones. Similar results were obtained in another study in which the author synthesized AgNPs and AuNPs with tyrosine and tryptophan [56].

Das et al. [57] demonstrated the ability of *Rhizopus oryzae* to prepare AuNPs and posited that the surface-bound protein molecules acted as both reducing and capping agents. This was later elucidated in detail by Kitching et al. [58], who prepared three different preparations of the cell surface proteins of *R. oryzae* and showed that the extracted surface proteins played a crucial role in the reduction of Au(III) to produce AuNPs. Similarly, Li et al. [59] used a protein extract from radiation-resistant *Deinococcus radiodurans* to synthesize Ag, Au, and bimetallic Ag–Au NPs. The functional groups of proteins involved in the reaction were investigated via Fourier-transform infrared microscopy, which showed that the hydroxyl, amine, carboxyl, and phospho groups of proteins were involved in the synthesis and capping of NPs. Previously, another study established that the extracellular synthesis of AgNPs by silver-resistant *Morganella* sp. was mediated by silver-specific proteins excreted by the cells during growth via the silver resistance machinery [60]. Several other researchers have also demonstrated the intracellular and extracellular synthesis of AgNPs capped with proteins of several fungal strains [61–63].

Arakaki et al. [64] identified Magnetite biomineralization (Mms) proteins from the *Magnetospirillum magneticum* AMB-1 strain, which contain dense carboxyl and hydroxyl groups and had the ability to bind iron. These proteins are essential for the organism to synthesize nanosized magnetite crystals. Later, it was shown that it is possible to control magnetite crystal size distribution and morphology in the presence of recombinant Mms6 protein [65]. Another study demonstrated the possibility of controlling the size and morphology of magnetite crystals using a peptide mimicking the Mms6 protein via a partial oxidation method [66]. Prozorov et al. [67] used the Mms6 protein for the shape-selective synthesis of cobalt ferrite NPs. The Mms6 protein used in this study was bound to poloxamers, allowing for controlled diffusion and crystal growth. The reducing ability of various proteins, amino acids, peptides present in the microorganisms has been harnessed by various studies to produce a variety of NPs. The commonly employed method of using cell extracts for production of NPs often credits the reduction of metal salts to the presence of these compounds in the extract, among other biomolecules. This mechanism also hints at the relation between ability of NP generation and heavy metal resistance of the microorganism. Same as enzyme-based mechanisms, there are numerous ways to employ the microbial proteins in NP synthesis, imparting flexibility to the synthesis process.

### Other mechanisms

The presence of polysaccharides, exopolysaccharides (EPS), and cell surface characteristics has also been shown to be important for NP biosynthesis. AgNPs from *Verticillium* sp. were found to form on the surface of the mycelium, and the authors conjectured that the first step in their synthesis was the trapping of Ag^+^ ions onto the surface of the fungal cells, which may occur due to electrostatic attraction between the negatively charged groups of enzymes present in the cell wall [68]. In another study, lactic acid bacteria (LAB) were used as reducing and capping agents in the synthesis of AgNPs. LAB are known to produce EPS, which may provide sites for the biosorption of Ag^+^ ions. These EPS consist of different sugars, and the reducing sugars present in them may function as electron donors for the conversion of Ag^+^ to Ag^0^. The authors also suggested that an increase in pH can lead to more competition between protons and metal ions for binding sites, and this increased pH could also catalyze the opening of ring-shaped monosaccharide structures to an open-chain aldehyde form, which in turn can act as a reducing agent. Moreover, the presence of heteropolymeric EPS on the cell surface might affect the localization of NPs among various *Lactobacillus* species [69]. Since then, the production of NPs using extracted EPS has also been demonstrated [70, 71]. Recently, EPS derived from a LAB exhibiting 97.77% similarity to *Enterococcus faecium* was used for generating AgNPs [72].

Cologgi et al. [73] studied the reduction of U(VI) to U(IV) by *Geobacter sulfurreducens* and demonstrated the importance of cell surface pili during the reduction process. In contrast to a pili-deficient mutant strain that exhibited mineralization in the periplasmic space, pili expression led to a greater rate and quantity of U(VI) biomineralization, and the reduced particles accumulated extracellularly. Pili length was found to increase the surface area available for redox reactions and U(VI) binding. The reduction in localized sections of the outer membrane and the permeation of U(VI) into the periplasmic space were attributed to the presence of *c*-cytochrome on the cell surface. The mechanism of *c*-cytochrome function in electron transport in *S. oneidensis* MR-1 during the reduction of Fe (III) oxide has also been explored. Four *c*-cytochromes (CymA, MtrA, MtrC, and OmcA), a trans-outer membrane and porin-like protein (MtrB), are involved in the electron transfer system of *S. oneidensis*. These constitute the Mtr pathway, which is responsible for electron transfer from the quinone pool to the surface of Fe (III) oxides present on the exterior. CymA, a member of the NapC/NrfH family of quinol dehydrogenases, oxidizes quinols and transfers electrons to redox proteins in the periplasm, thus serving as a gateway to the Mtr pathway. Later, MtrA and MtrB transfer these electrons across the membrane via the bacterial type II secretion system, where they reach MtrC and OmcA, which act as terminal reductases and reduce Fe (III) oxides [74].

Another interesting but underexplored way of NPs production from microorganisms is via biofilms [75]. Initially, the presence of nanosized colloidal metals was reported in the naturally occurring biofilms. An aerotolerant sulfate-reducing bacteria of the family *Desulfobacteriaceae* was found to have Zinc sulfide NPs (ZnSNP) of 2–5 nm aggregated into spherical shapes [76]. Analogously, Karthikeyan et al. [77] exposed biofilms of *Pseudomonas aeruginosa* PAO1 to gold chloride and found that at high concentrations (0.5–5 mM), intracellular and extracellular colloidal particles were observed. This observation was consistent with the fact that *P. aeruginosa* forms biofilms possessing increased heavy metal resistance as compared to the planktonic cells [78]. Recently, this ability of biofilm has been harnessed by some researchers to synthesize AgNPs [79, 80]. The authors of the study proposed that the electrochemically active bacteria present on the biofilm oxidized the sodium acetate acting as an electron donor, which in turn reduced the silver ions to elemental AgNPs. However, the presence of ZnSNP in natural settings also raises concern about the NP induced contamination of various environments such as organic wastes used as fertilizers [81]. As of now, a lot more research is required to understand the mechanism behind NP generation from biofilms and the risks biofilm-mediated, naturally occurring NPs pose to the environment.

Analyses of the current literature indicate that a diverse number of pathways and mechanisms are responsible for the generation of NPs via microbial routes. From these studies, it is apparent that different microorganisms use different approaches to synthesize nanomaterials. This interesting facet of microbe-based NP synthesis not only introduces more complexity to the biosynthesis process but also provides flexibility and adaptability when devising a synthesis protocol. However, more research is required to address the gaps and exploit microbial resources economically. With the current emphasis on the discovery of microbial dark matter, it is plausible that novel biosynthetic pathways and more efficient NP-producing organisms could be identified. A deeper understanding of the biosynthesis routes would also allow researchers to apply and harness microbe-mediated production processes more efficiently.

## Effect of physicochemical factors on nanoparticle synthesis

NP synthesis is strongly affected by the physicochemical conditions of the reaction. The rates of nuclei formation, development, and stabilization of NPs are regulated by various factors, such as pH, precursor concentration, reaction time, temperature, and other reaction conditions. A thorough understanding of the effects of these parameters is essential to efficiently control the synthesis process and aspect ratios of the produced NPs. The effects of these parameters on the synthesis of various NPs are discussed with selected studies.

### Effect of pH

The pH of the reaction mixture has a paramount effect on the NP synthesis rate, size, and stability. The pH possibly affects the enzymes and proteins responsible for synthesis and hence alters the structure and rate of NP formation. Several bacteria have been used to synthesize different types of metallic NPs in various pH ranges. Gurunathan et al. [82] studied the formation of AgNPs via *E. coli* at a pH range of 8–12, a 5 mM AgNO_3_ concentration, and at 60 °C. The authors observed a maximum synthesis at pH 10, and as the pH was further increased, the yield decreased. In addition, the mean NP diameter was the smallest at pH 10 (approximately 10–15 nm). The authors attributed the change observed in the reaction rate to the increased reducing power of proteins involved in the reaction under alkaline conditions. Similar results were achieved when AgNPs were synthesized from *Bacillus* sp. SBT8. The authors studied the reaction of AgNPs with cell-free supernatants at pH 6, 7, 8, 9, and 10 and showed that the highest absorption intensity was observed at pH 10. The surface plasmon resonance (SPR) peak at pH 8 was observed at 416 nm, which increased to 430 nm with an increase in the pH to 10, indicating the formation of larger NPs. The TEM observation showed that mean particle sizes at pH 8, 9, and 10 were 9.1 ± 5.3, 15.0 ± 10.7, and 28.2 ± 13.4 nm, respectively [83]. In another study, *Brevibacillus brevis* (formerly *Bacillus brevis*) culture supernatant-mediated AgNPs produced smaller particles at pH 9 than at pH 5 and 7.2 [84]. Another research group studied the synthesis of AgNPs from *Lysinibacillus sphaericus* MR-1 cell-free extract at various pH levels (6–13) and found that alkaline conditions increased the yield of the reaction, with maximum absorbance observed at pH 12 [85].

He et al. (2007) synthesized AuNPs from *Rhodopseudomonas capsulata* biomass and found that the pH was the most important parameter in controlling the size of the obtained NPs. The authors reported that transmission electron microscopy (TEM) measurements showed that at pH 7, the NPs were mostly spherical, with a size range of 10–20 nm. As the pH decreased to 4, several nanoplates of 50–400 nm were observed with spherical particles in the size range of 10–50 nm. The authors concluded that a change in pH regulates the proton concentration, and at a low pH, the functional groups responsible for the reduction carry a positive charge, which leads to the close binding of negatively charged ions and reduces the reduction speed. As the pH increased, the reducing power increased, giving rise to thermodynamically favored spherical shapes [86]. The effect of pH on the deposition of AuNPs in *S. algae* cells was elucidated, and the authors found that at pH 7, AuNPs of 10–20 nm in size were deposited in the periplasmic space of the organism, and as the pH was reduced to 2.8, the size of the NPs increased to 15–200 nm, with various morphologies were observed on the cell, such as triangular and hexagonal shapes. With a further reduction of the pH to 2, the size of intracellular AuNPs measured was ~ 20 nm, whereas that of the extracellularly deposited NPs was ~ 350 nm, suggesting that the enzyme responsible for extracellular reduction was released into the aqueous medium. The data showed that pH affected not only the size and shape of the NPs but also their translocation [87]. An actinomycete, *Gordonia amarae*, mediated AuNP synthesis in a pH-dependent manner. The synthesis was studied between the pH range of 4–12, and it was found that alkaline conditions at pH 10 and 12 were conducive for NP generation [88].

Sinha et al. (2011) studied a mercury-resistant strain of *Enterobacter* sp. that could bioaccumulate mercury with simultaneous synthesis of mercury NPs (HgNPs). To understand the effect of pH on the synthesis of HgNPs, cells were grown at different pH values in the presence of HgCl_2_. TEM images revealed particles of irregular shapes and sizes at pH 6, compared to uniformly distributed spherical NPs on the cell wall and inside the cell at pH 7, with sizes between 2 and 5 nm. Dense synthesis of intracellular NPs was observed at pH 8, and as the pH increased to 9, the particle size decreased with less dense synthesis [89].

**Fig. 3:**
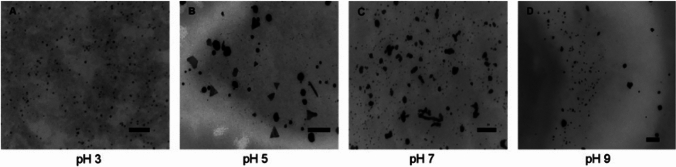
Effect of pH on gold nanoparticle morphology in *Verticillium luteoalbum* (scale bar = 100 nm). Adapted from [90], Springer Nature (CC BY 2.0.)

The effect of pH on the shape and size of NPs produced by fungi has also been demonstrated previously. *Verticillium luteoalbum* and an isolate obtained from soil and metal-rich dump samples, named Isolate 6–3, mediated synthesis of AuNP was studied at pH 3, 5, 7, and 9. The particles obtained at pH 3 were uniform, spherical, and predominantly less than 10 nm in size (Fig. 3). At pH 5, the spherical particles were accompanied by larger hexagonal, triangular, and rod-shaped particles. As the pH was further increased to 7 and 9, small spherical particles were present, including particles with larger, irregular, and undefined shapes (Fig. 3) [90, 91]. The yeast, *Yarrowia lipolytica* NCIM 3589, was used to produce AuNPs at pH 2, 7, and 9, and the reduction observed was likely influenced by the pH. Under acidic conditions, nucleation occurred on the cell surface, leading to the formation of large crystals. No crystal formation was observed at a higher pH, and 15-nm-sized particles were found to be organized on the cell wall [92]. Similarly, the AuNPs synthesized by the intracellular protein extract of *Pycnoporus sanguineus* showed a maximum SPR between pH 4 and 6, but the size decreased with an increase in pH. At pH 2.0, the AuNP size ranged from a few nanometers to 200 nm, with an average particle size of 84.29 nm. Smaller particles were observed with an increase in pH to the neutral or basic range. At pH 12, most particles were smaller than 12 nm in size. In addition, the color of the reaction mixture at pH 12 changed to a bluish violet within 2 h, whereas this occurred at pH 4 and 2 after a longer period (4 and 8 h, respectively). This indicates that the reaction rate increases with an increase in pH [93]. *Trichoderma viride*-synthesized AuNPs showed a decrease in particle size with an increase in pH, along with changes in the shape of the particles. At pH 5.0, various shapes, such as large prisms, triangles, pentagons, hexagons, and rods, were synthesized with sizes ranging from 5 to 200 nm. As the pH increased, the shape of the particles changed until pH 9, at which the particles became small (approximately 3–10 nm), with a spherical shape. A further increase in the pH changed the particle shape to an irregular one. This observation was attributed to a decrease in repulsion that occurs between negatively charged precursor molecules and carboxylic functional groups at a low pH, which leads to unrestrained nucleation and the consequent formation of large, mixed-shape particles [94].

Nayak et al. (2011) utilized *Penicillium purpurogenum* NPMF culture filtrate to produce AgNPs and found that the synthesis and size of the particles were dependent on the filtrate pH. The pH was varied from 4 to 9, and the SPR at approximately 420 nm was observed at pH 7, 8, and 9, along with the original pH (8.3) of the filtrate. The SPR for pH 4, 5, and 6 shifted to higher wavelengths, indicating polydispersity and a larger size. TEM images confirmed the change in size observed due to pH, as 40–55-nm particles were observed at pH 4 and 5, whereas the sizes of the particles formed at pH 8 and 9 were in the range of 8–13 nm. The authors hypothesized that proton concentration affected the nitrate reductases present in the filtrate, thereby changing the morphology and size of the particles [95]. A recent study on AgNP production by the endophytic fungus, *Phyllosticta capitalensis* Henn., used RSM to statistically optimize NP production in terms of size and maximum wavelength by optimizing several physicochemical parameters: pH, reductant concentration, precursor-to-reductant volume ratio, and reaction time. The results indicated the dominance of pH, pH-linked interaction effects, and square effects on AgNP synthesis. The model predicted the optimal NP size as 105.86 nm and wavelength maxima as 413.5 nm [96].

AgNP, copper-oxide NPs (CuO NP), and iron-oxide NPs (FeO NP) were synthesized via aqueous extracts of *Botryococcus braunii* and the effect of physicochemical parameters was examined. The synthesis was carried out at various pH from 3 to 11. For AgNPs and FeO NP synthesis, pH 11 was found to be optimal, whereas for CuO NP, smallest particles were obtained at pH 9. Under initial reaction conditions of precursor concentration (50%) and incubation temperature (25 °C), the size range of AgNPs synthesized under different pH conditions were from 66.94 ± 7.92 to 96.34 ± 6.73 nm. Compared to the other factors, pH affected the NP size most strongly. After optimization, the smallest sizes of AgNPs, FeO NPs, and CuO NPs obtained were 43.46 ± 6.38 nm, 62.86 ± 10.08 nm, and 70.78 ± 8.96 nm, respectively [97]. Riddin et al. [98] demonstrated the importance of pH in the production of platinum NPs from *F. oxysporum* f. sp. *lycopersici*, along with other physicochemical factors. Using RSM, the researchers were able to optimize the yield, with temperature and pH playing an important role in the shapes of NPs produced. These studies demonstrated the effects of pH on the synthesis rate, size, and shape of NPs. Several cases seem to be leaning toward the use of neutral or alkaline pH; however, the optimum pH should always be determined in order to control the biological synthesis processes.

### Effect of temperature

The effect of temperature on microbe-assisted synthesis of NP is a vital parameter because the reaction can occur from ambient to high-temperature conditions, covering a wide temperature range. This makes it important to understand the optimum temperature required in each case to ensure an optimal reaction rate along with the control on the size of the NPs produced. Rajendran and Sen [99] analyzed the effect of temperature on the formation of hematite NPs by the culture supernatant of *Bacillus cereus* SVK1, using treatment temperatures of 40 °C and 30 °C. The authors concluded that NP formation occurred rapidly at these temperatures; however, when the temperature was increased above 40 °C, no reaction was observed, with 37 °C being the optimum temperature. It was hypothesized that the latter occurs because of the inactivation of biomolecules that reduce the ferric chloride solution. A previous study that synthesized AgNPs from a *Bacillus* sp. strain suggested that temperature changes influenced the yield, and maximum synthesis was observed at temperatures of approximately 33–37 °C. The authors also concluded that even though the absorbance was low at 30 °C, the morphology and size of the NPs detected via scanning electron microscopy were similar to those at 33 °C. However, the formation of NPs reduced considerably at 40 °C, and the average particle size was 20.7 ± 10.5 nm at 33 °C [83]. In contrast, Patil et al. [100] observed the impact of temperature on AuNP synthesis using a novel marine bacterium and showed that reactions at 60 °C and 70 °C exhibited broad and sharp peaks, respectively; however, no peak was observed at temperatures of 30, 40, and 50 °C. The authors also mentioned that an increase in the reaction temperature resulted in an enhanced reaction rate and the formation of small, monodispersed AuNPs. Another study by Bennur et al. [88] observed the role of temperature during NP synthesis via the actinomycete, *G. amarae*. As per the study, no distinctive peak at 530 nm was observed at 60 °C; however, upon a temperature increase (to 80–90 °C), a distinct ruby-red color in the reaction mixture was observed, indicating the presence of AuNPs.

The effect of temperature on NP synthesis has been studied in several fungal species. Mohammed Fayaz et al. (2009) conducted a study where an increase in the reaction temperature led to a reduction in the size of AgNPs synthesized via *T. viride*, and the morphology and uniformity of AgNPs were controlled by temperature. The authors studied the effect of reaction temperature by maintaining a neutral pH and observed that absorbance peaks were obtained at 405 nm (40 °C), 420 nm (27 °C), and 451 nm (10 °C), the characteristic plasmon band for AgNPs. Sharp narrow peaks were observed at 40 °C, corresponding to small-sized NPs, and the shift in the peaks found at lower temperatures indicated an increase in the size of AgNPs [101]. Mariekie Gericke and Pinches (2006) described the role of temperature in the formation of AuNPs via *V. luteoalbum* and their morphology. Most of the NPs formed after exposure to gold solution for 1 h were spherical, with an average diameter of 10 nm at lower temperatures of 25–30 °C. However, upon further incubation for 24 h, the number of large particles with well-defined shapes increased. At 50 °C; however, no difference could be detected in the size and morphology of particles produced after 1 and 24 h of exposure, with very few small, spherical particles present. The study speculated that the optimum temperatures for cell growth and gold accumulation are different. The rate of NP formation depended on the incubation temperature, and increased temperature levels allowed for a faster particle growth rate [90]. Husseiny et al. [102] studied the biosynthesis of size-controlled AgNPs by *F. oxysporum* and observed that the incubation temperature plays a vital role in NP synthesis. The AgNP size decreased with an increase in temperature until it reached the smallest size of 30.24 nm at 50 °C. However, upon incubation at temperatures higher than 50 °C, the particle size increased due to the denaturation of enzymes. Another study conducted to evaluate the growth of AgNPs from various isolates of *F. oxysporum* stated that an increase in incubation temperature increases the quantity of NPs, with the NP diameter having an inverse relationship with temperature. The smallest NP size and highest yield were obtained at 75 °C. The authors estimated the quantity of AgNPs produced at different temperatures by considering the NP size and corresponding extinction coefficient and concluded that higher concentrations of AgNPs were produced at higher temperatures [103].

Koçer and Özçimen (2023) studied the effect of temperature on biosynthesis of AgNP, CuO NP, and FeO NP from the aqueous extracts of *B. braunii*. The synthesis was carried out at 25 °C, 40 °C, 55 °C, 70 °C, and 85 °C for 1 h, at optimal pH and metal solution concentration. For AgNP, the particle size decreased when the temperature was increased from 25 to 55 °C and increased with temperature increase thereafter. The optimal temperature for FeO NP and CuO NP synthesis were found to be 70 °C and 55 °C, respectively; however, temperature variation did not impact the particle size strongly [97]. This temperature information clearly shows the variations in optimum temperature required for the different NP generation. The reaction temperature seems to be specific to the organisms, enzymes, proteins, and synthesis processes; therefore, it is an important factor that needs to be optimized to alter the yield, rate of reaction, and morphology of NPs.

### Effect of reaction time

**Fig. 4:**
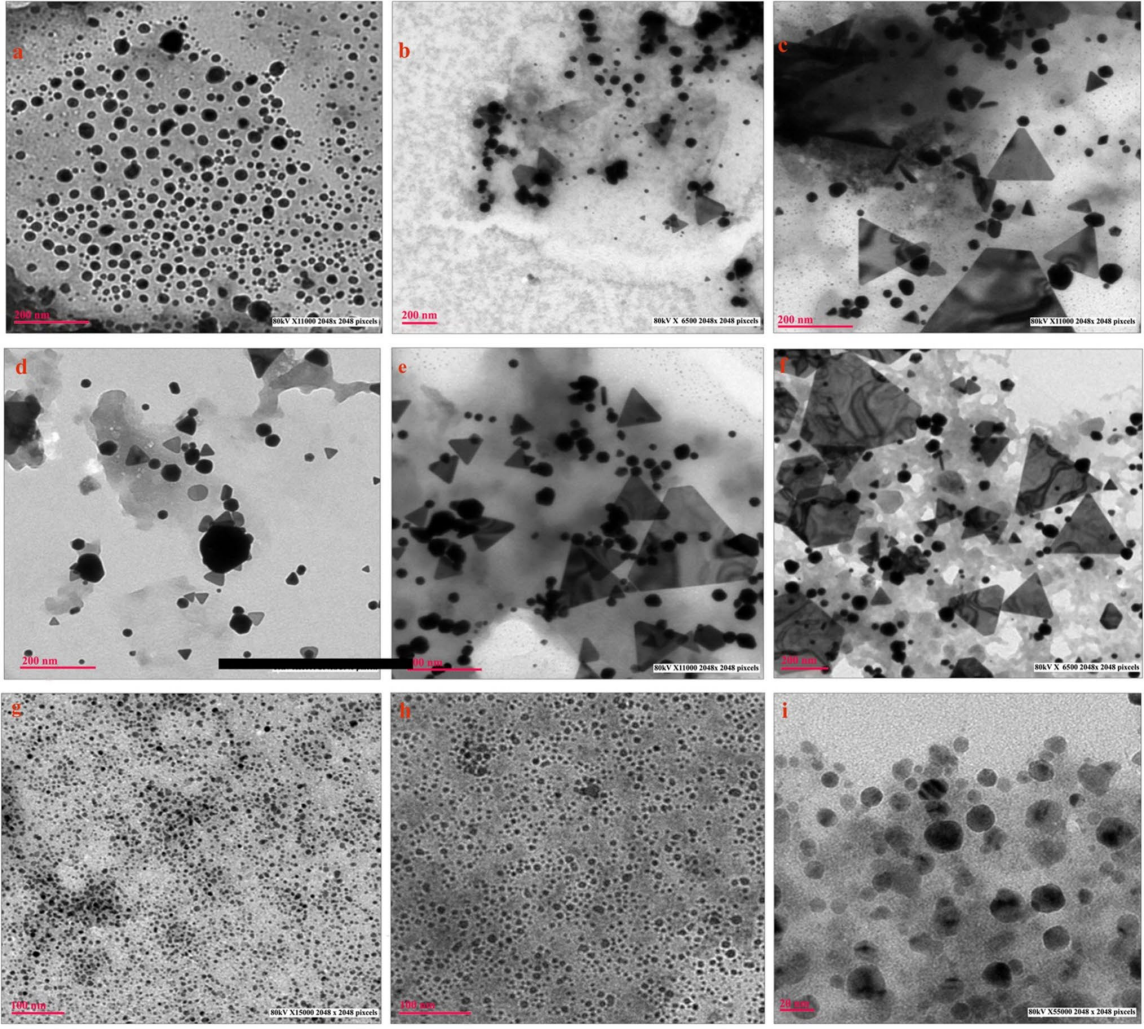
Effect of reaction time and pH on the shapes and sizes of gold nanoparticles synthesized by *Trichoderma viride* filtrate. At pH 5 after **a** 24 h, **b** 48 h, and **c** 72 h. At pH 7 after **d** 24 h, **e** 48 h, and **f** 72 h. At pH 9 after **g** 24 h, **h** 48 h, and **i** 72 h. Adapted from [94], Springer Nature (CC BY 4.0.)

The reaction time for NP generation in microbe-mediated synthesis generally refers to the time of contact between the precursors and reducing agents. The reaction time can affect the yield and size of the NPs produced. A study by Patil et al. (2019) observed the effect of incubation time on the production of AuNPs using the marine bacterium, *Paracoccus haeundaensis* BC74171T, by running the reaction at a constant temperature of 70 °C at varying incubation durations. The authors observed an increase in absorbance with an increase in incubation time, thus increasing the number of NPs produced. They also suggested that an optimum reaction time of 15 min was preferred for the maximum production of AuNPs [100]. Husseiny et al. (2015) investigated the effect of fungal biomass age along with varying incubation times using *F. oxysporum* and observed that the size of NPs decreased when seven-day-old fungal biomass was used. However, using 15-day-old biomass increased the size of the produced AgNPs. The authors concluded that the maximum yield with the smallest particle size was achieved after 72 h of incubation when the fungus was seven days of age [102]. Kumari et al. (2016) studied the effect of reaction time on the synthesis of AuNPs using *T. viride* filtrate and observed a large impact on the morphology of AuNPs formed at 24, 48, and 72 h at pH 5.0, 7.0, and 9.0. The authors suggested that after 24 h, 100% of the synthesized particles were small spheres (7–24 nm), which might be due to initiation of the nucleation process, gradually followed by the presence of a mixed population of spheres, triangles, and prisms of larger sizes (7–120 nm) after 48 h, owing to crystal growth. As the reaction progressed beyond 72 h, the particles formed were predominantly triangles and prisms of 20–400 nm in size. Interestingly, the shapes and sizes were unaffected at higher pH for long durations, resulting in no changes in the geometry and dimensions of the biosynthesized NPs, even after 72 h (Fig. 4) [94]. Kathiresan et al. [104] conducted studies on AgNPs using the marine fungus, *Penicillium fellutanum*, and observed the effect of incubation time on NP synthesis from 0 to 48 h. The results showed that the highest optical density (OD) was obtained after 24 h of incubation, after which the OD decreased. Basiratnia et al. [105] studied the effect of contact time on NP synthesis using green microalgae and showed that an increase in the reaction time resulted in an increase in intensity of the SPR band, with no shift in wavelength observed, indicating a rapid increase in the number of AuNPs produced. The authors conducted the study for six days, and the reaction between Au^+^ ions and reducing groups of the extract was observed. The AuNPs synthesized through this reaction were quite stable and did not aggregate even after 4 months.

### Effect of reactant concentration

Reactant concentrations, such as the precursor concentration, the cell extract/supernatant volume, and concentration, also play an important role in the synthesis of NPs. These factors are important in biosynthesis because active components in the cell extract or culture supernatant are limited by design. Optimum reactant concentrations allow for the nucleation and proper development of NPs. If the volume or concentration of the reactions are incorrect, this might lead to the aggregation of particles, possibly due to the lack of reducing and stabilizing groups. Gurunathan et al. (2009) studied the effects of AgNO_3_ on the reaction rate and particle size of AgNPs synthesized in the extracellular supernatant of *E. coli*. NP synthesis was studied from 1 to 10 mM AgNO_3_, and the results showed that the absorption maxima observed at 420 nm increased up to 5 mM and decreased thereafter. The size of the synthesized NPs decreased as the precursor concentration increased from 1 to 5 mM, reaching a size of 15 nm, and increased thereafter. The authors speculated that the decrease in size observed up to 5 mM could be due to AgNO_3_, which forms a coat on growing particles, preventing their aggregation and reducing their size, and that the dispersive action of silver ions might be responsible for controlling the size of AgNPs [82]. AuNP production from the actinomycete, *G. amarae*, was maximal at a precursor concentration of 2 mM [88]. The use of *Bacillus* sp. SBT8 to produce AgNPs was affected by the concentration of AgNO_3_. The results showed that concentrations of 4 and 6 mM were optimal for NP production, and no SPR peak was observed at higher concentrations. The average sizes obtained at pH 4 and 6 were 11.9 ± 6.2 and 9.1 ± 5.3 nm, respectively [83]. Patil et al. (2019) studied the effects of different volumes of cell extracts and various concentrations of HAuCl_4_ on NP formation in *P. haeundaensis*. The NP yield increased as the cell extract volume was increased from 2 mL (no yield) to 10 mL (maximum yield), as well as when the HAuCl_4_ concentration was increased from 0.25 mM (minimum yield) to 2 mM (maximum yield), without any shifts in the peak observed [100]. Production of AgNPs using 50 mL cell filtrate of fungi *P. fellutanum* with 0.5–2.5 mM AgNO_3_ showed that 1 mM was the optimum substrate concentration required. Moreover, the authors considered the effect of salinity on AgNP production and found that 0.3% NaCl was optimal [104]. In contrast, Nayak et al. (2011) showed that after 24 h of AgNP synthesis using *P. purpurogenum* NPMF cell extract, the absorbance maxima at 420 nm increased with increasing AgNO_3_ concentration up to 5 mM; however, the TEM images indicated that as the precursor concentration increased, so did the polydispersity. At 1 mM, monodispersed particles with an average size of 10 nm were observed, whereas at 5 mM, irregularly shaped particles with polydispersity were observed [95]. Mariekie Gericke and Pinches (2006) demonstrated the effect of increasing AuCl_4_^−^ ion concentrations on the production of AuNPs via *V. luteoalbum* biomass. To study the effect of gold ion concentrations, cells were exposed to concentrations of 250, 500, and 2500 mg/L. TEM images showed the presence of small particles, and some large, spherical particles were spread throughout the cells at concentrations of 250 and 500 mg/L. As the concentration increased to 2500 mg/L, cells exhibited the presence of very large, irregular particles spread throughout [91].

Roy et al. (2016) studied AuNP formation mediated by *Aspergillus foetidus*, as well as the impact that variations in cell extract and precursor concentrations has on AuNP formation. The authors showed that undiluted and 10%-diluted cell extracts could produce the highest amount of AuNPs, demonstrating the importance of the ratio of the cell filtrate and metal salt concentration. The appropriate HAuCl_4_ concentration used in this study was found to be 1 mM, and as the concentration was increased to 5 mM, the polydispersity increased, as indicated by the broad UV–Vis spectrum [106]. In a similar study, Shi et al. (2015) investigated the effects of intracellular protein extract and initial gold ion concentration on the production of AuNPs from *P. sanguineus*. The authors found that as the volume of the intracellular extract increased from 10 to 80 mL, a blue-shift was observed, signifying the generation of small-sized NPs. This observation was corroborated by TEM images, where the 10-mL extract exhibited triangular or hexagonal shapes in the size range of 100–500 nm, with a mean of 61.47 nm. When the extract volume reached 80 mL, the majority of particles were less than 50 nm in size, with an average particle size of 29.3 nm. The opposite effect was observed for the gold ion concentration. At a concentration of 0.5 mM, the NPs obtained were smaller and mostly spherical, with an average size of 25.88 nm. When the concentration increased to 2 mM, the average size increased to 51.99 nm. The authors explained that as the concentration of ions increased, accessibility of the formed gold atoms to the gold nucleus also increased, thereby enlarging the size of the AuNPs [93]. Recently, AuNP synthesis mediated by *Phormidesmis communis* strain AB_11_10 was elucidated. The synthesis protocol showed that an increase in the precursor concentration from 0.5 to 2 mM caused a red-shift from 533 to 562 nm, indicating the formation of NPs with large sizes or NP aggregates. A similar increase in the precursor-to-cell extract ratios from 1:1 to 1:2, 1:4, and 1:9 increased the SPR maxima from 533 to 542.5, 559.2, and 600 nm, respectively [107].

**Fig. 5:**
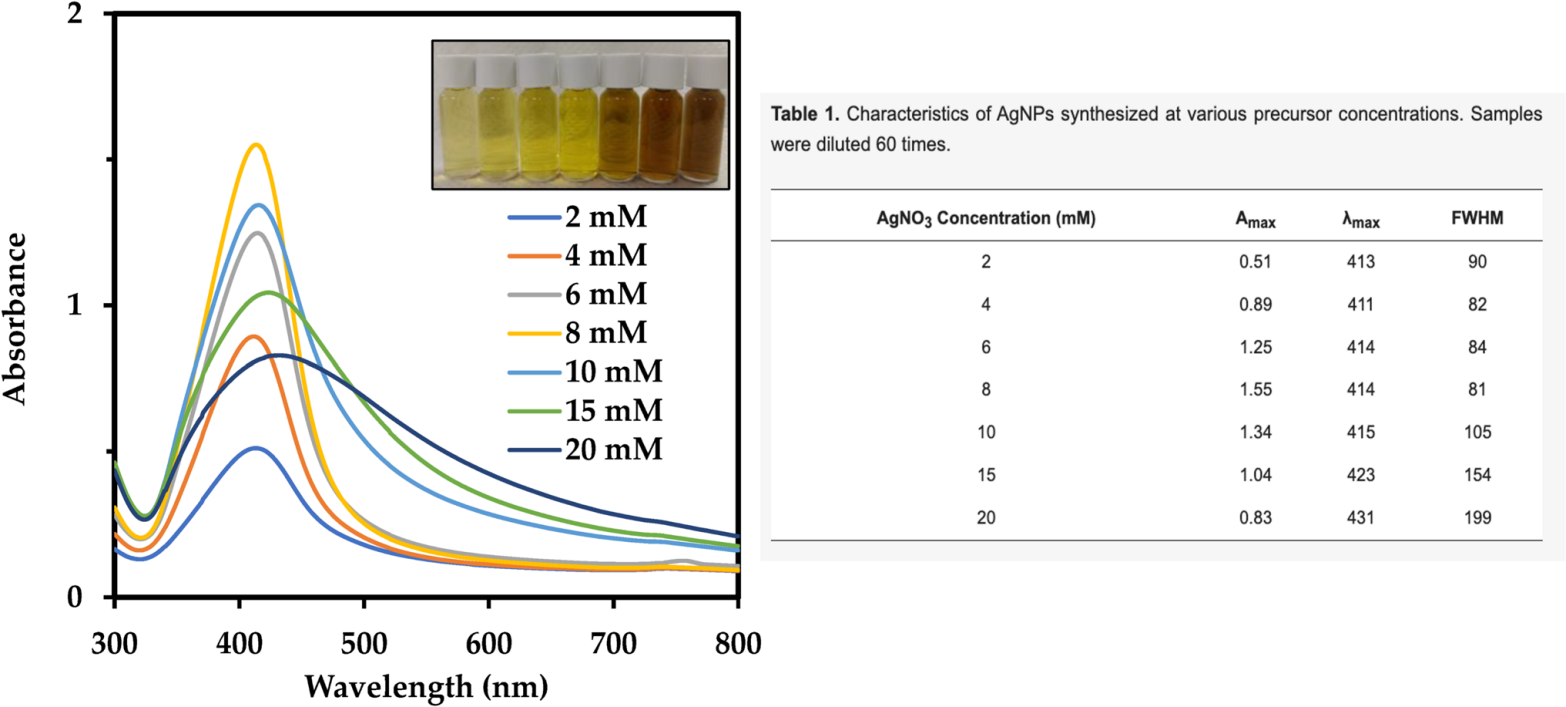
Effect of precursor concentration on the surface plasmon resonance and UV–Vis spectrum of silver nanoparticles (AgNPs) synthesized by *Talaromyces purpurogenus* extracellular pigment. The A_max_, λ_max_, and FWHM in the table refers to maximum absorbance, maximum wavelength, and full width at half maximum, respectively. Adapted from [110], MDPI (CC BY 4.0.)

Rose et al. (2019) investigated the effects of AgNO_3_ and the amount of biomass on AgNP synthesis via *P. oxalicum* GRS-1 cell extract. The results showed that NP production increased up to 1.5 mM AgNO_3_, and a further increase in the precursor concentration led to the peak distorting and flattening, indicating the formation of larger NPs. Biomass studies showed that as the biomass increased from 5 to 25 g, NP formation increased concurrently. The authors reasoned that at a lower precursor concentration, the lack of substrate availability for the enzyme led to a low production of AgNPs. The increase in productivity with increasing biomass was attributed to the presence of higher amounts of nitrate reductase [108]. Similar results were obtained by Gade et al. [109], where the production of AgNPs by the fungus, *Phoma glomerata*, was affected by the precursor concentration and amount of fungal filtrate used. The optimum precursor concentration was 0.8 mM, and the rate of synthesis of AgNPs increased with the filtrate volume. AgNP synthesis from the extracellular pigment of *Talaromyces purpurogenus* was affected by the AgNO_3_ concentration, where the best yield was obtained at 8 mM when the precursor concentration was varied from 2 to 20 mM (Fig. 5). Differences in the yield and SPR parameters were observed visually and by using UV–Vis spectrophotometric data [110]. Recently, the effect of chloroauric acid concentration on the synthesis of AuNPs using cell extract from the same organism was demonstrated. A 0.6-mM precursor concentration was found to be optimal for AuNP synthesis. Above this concentration, the AuNP spectrum was red-shifted and unstable during storage, indicating a lack of stabilizing agents present [25].

The effect of metal salt solution ratio on the particle size was studied for AgNP, CuO NP, and FeO NP synthesis via cell extract of *B. braunii*, with precursor concentration ranging from 10 to 90%. The data indicated that at optimal pH conditions and 25 °C incubation temperature, the particle size was directly correlated with precursor concentration, and the optimal concentration was determined to be 10%. The authors concluded that with the increase in metal salt ratio, the size and aggregation of the NPs also increases due to increased interactions [97]. Sinha and Khare [89] reported that the initial HgCl_2_ concentration affects the intracellular accumulation of HgNPs by *Enterobacter* sp. The production of nickel oxide NPs by the dead fungal biomass of *Hypocrea lixii* showed that, among the three types of biomasses used in the study (live, dead, and dried), dead biomass was more effective at removing nickel than live and dried biomasses were, indicating a higher affinity of dead biomass toward nickel. The removal of nickel was also found to be dependent on the amount of biomass and initial concentration of nickel used [111]. The *B. cereus* SVK1-mediated hematite NPs were affected by the type of precursor, precursor concentration, and culture supernatant-to-precursor ratio concentration. Among the tested precursors, ferric chloride reduced the fastest, after 21 days. Solubility of the precursors was credited to differences in kinetics of the various reactions. As the concentration of the precursor increased, the time required for reduction also increased. This was attributed to the presence of insufficient amounts of biomolecules required for reduction and stabilization. Increasing the supernatant-to-precursor ratio increased the rate of formation of hematite NPs [99]. In a recent study, the volume of algal extract from *Chlorella* sp. was shown to affect the phase transformation of titania NPs. The diffraction data showed that when 80 mL of 0.5 M precursor was reduced using 5–15% (v/v) algal extract, the end product contained mixed phases of anatase, brookite, and rutile, whereas increasing the extract to 20% led to the production of anatase and brookite phases, and the phase transformation from anatase to rutile was inversely related to the algal extract volume [14]. Various studies involving the precursor concentration and reducing agent volume/concentration have shown the importance of these factors in the microbial synthesis of NPs and these values should be chosen carefully to optimize the reaction kinetics, shape, and size of NPs.

### Effect of light

Light is known to affect NP synthesis by modulating the reaction rate or altering the shape and size of the produced NPs. Shivaji et al. [112] used five psychrophilic and two mesophilic bacteria for the synthesis of AgNPs under the light of a 9 W compact fluorescent lamp, as well as under dark conditions, and showed that AgNP formation occurred only in the presence of light. In another study, *P. glomerata* fungal extract-mediated AgNP generation showed the production of NPs under the influence of sunlight; the authors also investigated the effect of various light intensities on the production of AgNPs, along with sunlit and dark conditions. The results demonstrated that AgNP synthesis was the highest under sunlight, followed by tubelight (190.7 lx), bulb light (141.39 lx), and moonlight (15.5 lx). Synthesis was evident in the presence of sunlight in only a few minutes, whereas it took a few hours under tubelight and bulb light conditions. The authors asserted that the effect of light can be explained by the photosensitization of aromatic molecules present in the filtrate, and that the free electrons generated due to this were responsible for the reduction observed. The authors also showed that synthesis was optimal under blue light, and concluded that under blue light irradiation, fungal proteins serve as photoreceptors, and proteins consisting of flavin-binding motifs can be sensitized [109]. A similar conclusion was reached by Neethu et al. (2018), who synthesized AgNPs from *Penicillium polonicum* in the presence of light (40 W, 2400 lumens) and observed a signature color change to dark brown within 60 min, whereas the sample under dark conditions slowly turned light brown. The authors concluded that the reduction of silver ions was a synergistic process involving fungal filtrate proteins and light. They also alluded to the photosensitization of aromatic compounds present in the fungal filtrate in the presence of light [113]. Cell-free filtrates extracted from *F. oxysporum* grown in the presence and absence of light showed that the extract from the cells grown under exposure to a halogen lamp had better productivity than the extract from cells grown in the dark. However, this difference was attributed to the secretion of higher amounts of nitrate reductase enzyme under irradiated conditions. Nitrate reductase production was induced in the presence of light, which consequently increases NP production [37]. Recently, *Saccharomyces cerevisiae* DSM 1333 cellular extract was used in conjunction with light irradiation to control AgNP synthesis. The NP yield was augmented by 90% and 70% after exposure to 1.0 ± 0.2 and 0.5 ± 0.1 mW/cm^2^ light irradiation, respectively. The synthesis was further optimized by considering yeast cultivation conditions (aerobic and oxygen limited), filtrate extraction temperature, synthesis temperature, and precursor concentration [114]. Zhang et al. (2016) studied the effect of light on AgNP synthesis via EPS derived from *S. oneidensis* MR-1. The reduction of silver ions under the presence of light (500 μW/cm^2^, 400–800 nm) was characterized as having pseudo-first order kinetics. The authors also investigated the effects of simulated sunlight (290–800 nm), visible light with a 400-nm cutoff filter (400–800 nm, 500 μW/cm^2^), and UV light (290–400 nm, 70 μW/cm^2^) on AgNP synthesis. AgNP formation was fastest under UV light (rate constant, *k* = 0.188 h^−1^), followed by simulated sunlight (*k* = 0.174 h^−1^) and visible light (*k* = 0.143 h^−1^). Visible and UV light had no effect on Ag^+^ ions in the absence of EPS. No SPR peak was detected in the dark for up to 60 h but could be detected after 14 days. These results imply that light stimulates the reduction of silver ions via EPS. The authors assumed that the effect of visible light was due to autocatalysis of the produced NPs, similar to the photodeposition of noble metals. The authors also surmised that interaction with UV light led to the formation of species with strong reducing capabilities, such as hydrated electrons and reducing radicals, responsible for Ag^+^ reduction [115]. The effect of light on AgNP generation has been previously elucidated by using *P. oxalicum*, *Klebsiella pneumoniae*, and *B. amyloliquefaciens* [116–118].

**Fig. 6:**
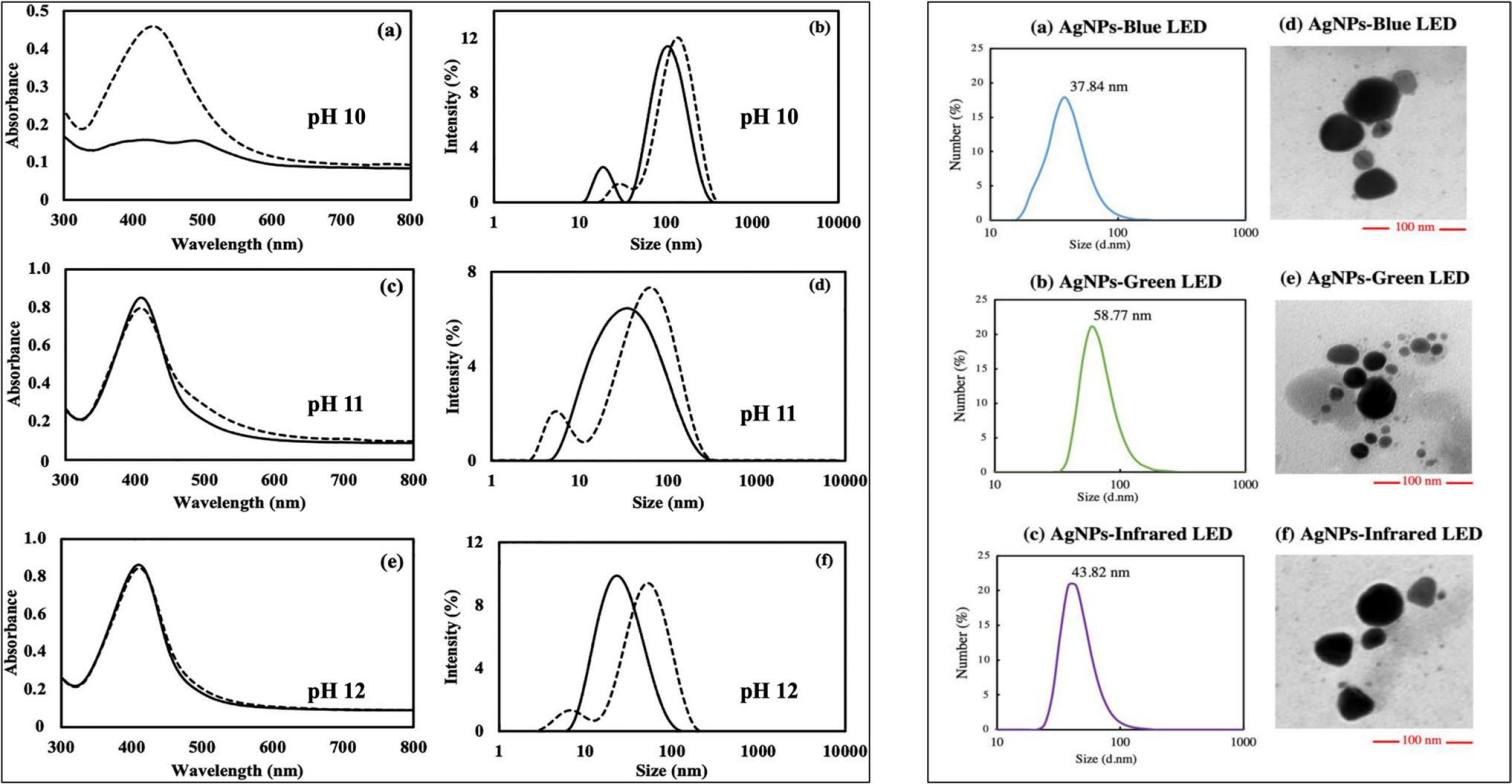
Effect of visible light (2000 lx) and pH on the synthesis and size distribution of silver nanoparticles (AgNPs) synthesized from the extracellular pigment of *Talaromyces purpurogenus* (left panel). Dashed and solid lines represent light and dark conditions, respectively. Adapted from [120], Taylor and Francis (CC BY 4.0). Effect of LED lights on biosynthesis at pH 10 from the extracellular pigment of *T. purpurogenus* (right panel). Adapted from [121], MDPI (CC BY 4.0.)

Pigments produced by fungi have been used in conjunction with light sources to generate AgNPs [119]. Bhatnagar et al. (2019) reported the synthesis of AgNPs via extracellular pigments produced by *T. purpurogenus* in the presence of light. This synthesis mechanism was further explored in terms of the concerted effects of light and pH [120]. The yield from the synthesis was found to be dependent on the presence of light (2000 lx) up to pH 10, and the difference in yield was apparent when using UV–Vis spectrophotometry. Increasing the pH further led to a rapid increase in the reaction rate, and the synthesis was governed by pH, with little difference in the yield observed between light and dark conditions. Intriguingly, dynamic light scattering data indicated that the AgNPs synthesized in the dark were smaller under all monitored pH conditions (Fig. 6). The authors concluded that the addition of NaOH to increase the pH accelerated the process, and at sufficiently high concentrations, the effect of pH dwarfed the effect that light had on the synthesis process. This effect of light was further explored by Nuanaon et al. (2022), by using various light-emitting diodes (LEDs) to synthesize AgNPs. Exposure of the reaction mixtures to blue, green, and infrared LEDs significantly increased the AgNP yield (Fig. 6). This effect was ascribed to the higher energy of electromagnetic radiation, and the results reflected this, as the yield was found to be maximum after blue LED exposure, followed by green, orange, and red LED exposure. However, exposure to infrared LED was also found to augment AgNP synthesis [121].

Microalga *Neochloris oleoabundans* cell extract was used with a 5000 lx fluorescent light for AgNP synthesis and showed that 1 mM AgNO_3_ could be reduced in the presence of light, but not in the dark. Another study demonstrated the possibility of AgNP biosynthesis under white, blue, or purple light illumination in *N. oleoabundans*. Researchers have reported that light-excited chlorophylls donate electrons, reducing Ag^+^ to Ag^0^ in the process [122, 123]. In a recent study, cell extracts of the cyanobacterium, *Oscillatoria limnetica*, were used to synthesize AgNPs at three different light intensities: 57.75, 75.90, and 1276.51 μmol m^−2^ s^−1^. The results indicated that maximum absorption peaks were detected after exposure to sunlight (1276.51 μmol m^−2^ s^−1^), followed by 75.90 and 57.75 μmol m^−2^ s^−1^, and the lowest absorption peak measured in the dark. The results showed that the presence of light increased the rate of AgNP formation, and the authors concluded that synthesis in the dark might be dependent on proteins, soluble sugars, polysaccharides, and reductases, whereas light-harvesting pigments, intracellular components, proteins, and carbohydrates play roles in the presence of light [124].

## Effect of physicochemical factors on the applications of nanoparticles

As discussed previously, physicochemical factors affect the size, distribution, morphology, stability, and surface properties of NPs. These properties can have a profound effect on the application of NPs. Some studies in which the physicochemical factors were controlled to tailor particles for suitable applications are discussed herein. Nayak et al. (2011) varied the precursor concentration and pH of *P. purpurogenum* NPMF cell extract and evaluated the antimicrobial activity of the formed particles against *P. aeruginosa*, *E. coli*, and *Staphylococcus aureus*. They found that the NPs formulated under different conditions exhibited different zones of inhibition. This effect was attributed to the production of particles of different sizes and morphologies under different conditions [95]. In the chemical-based synthesis from sodium borohydride and polyvinyl alcohol, the size of the AgNPs was controlled by varying the pH (6, 8, 10, and 12) using NaOH. The resultant AgNPs showed a clear variation in crystalline size, as determined using X-ray diffraction (XRD), with particles of approximately 31 nm at pH 6, which decreased to 14 nm at pH 12. TEM images and SPR maxima confirmed this observation. The TEM images showed a decrease in particle size from 26 to 10 nm as the pH increased from 6 to 12. The zone of inhibition caused by the particles during disk diffusion test with *E. coli*, *Pseudomonas* sp., *A. niger*, and *Penicillium* sp. increased with increasing synthesis pH. In all cases, the maximum zone of inhibition was observed at pH 12. This phenomenon was attributed to the decrease in size observed, which increased the permeation ability of the NPs produced [125]. In another report, AgNPs were biosynthesized in *B. brevis* culture supernatant at pH 5, 7.2, and 9. Various volumes of colloidal solutions generated at these pH ranges were used to test antimicrobial activity against *E. coli*. The maximum zone of inhibition was observed at pH 9 and a volume of 50 μL. The authors reasoned that at a low pH, larger particles were synthesized, leading to a lack of potent antimicrobial activity [84].

Ocsoy et al. [126] synthesized AgNPs using red cabbage extract combined with a hydrothermal synthesis approach to control the yield and size distribution of NPs. They then applied NPs produced at different reaction times (15, 30, and 60 min) with different volumes of the extract (1 and 5 mL) to assess antimicrobial activity against *S. aureus*, *E. coli*, and *Candida albicans*. Although complete inhibition was achieved at high concentrations in all cases, the tests conducted at 75 and 37.5 ppm revealed that the antimicrobial activity increased with increasing synthesis time. A similar result was obtained using 5 mL of the extract. It was assumed that a longer incubation period led to the formation of smaller NPs, and that the formed NPs held a higher number of active atoms along with localization of the extract on the surface acting in conjunction with AgNPs [115]. Manosalva et al. [127] prepared AgNPs of different sizes (23, 92, and 220 nm) by varying physicochemical synthesis parameters and found that antimicrobial activity was inversely correlated with size. Riaz et al. [128] tested the collective effect of the pH of tea extract and temperature on antimicrobial activity and found that the pH and temperature regimes resulting in smaller NPs coincided with a maximum antimicrobial activity. In a study on the effect of calcination temperature on microbially synthesized AgNPs, particles were synthesized and later subjected to heat treatments of 100 °C or 300 °C for 2 h for crystallization. The results showed that heat-treated particles exhibited no antimicrobial activity, in contrast to the untreated particles. This was due to an increase in particle size to 400–800 nm compared to the 10–20-nm NPs formed without heat treatment [129]. Size-dependent antimicrobial activity has also been observed for PdNPs, where smaller, green-synthesized particles exhibited higher antifungal activity [130]. The effect of heat treatment on CuO NPs generated via a reflux method showed that the particles exposed to 800 °C resulted in smaller sizes with significant antimicrobial activity [131]. The effect of AgNP size on uptake and toxicity in human cell lines (A549, SGC-7901, HepG2, and MCF-7) has also been studied. Smaller particles were found to enter cells more easily, possibly causing increased toxicity [132].

Along with size, surface morphology has also been shown to affect the catalytic ability of the NPs. Kumari et al. [94] obtained different morphologies of AuNPs such as spheres, triangles, penta/hexagons, and sheets by modifying the physicochemical parameters while using *T. viride* cell-free extract for synthesis. The different types of AuNPs were used to degrade 4-nitrophenol, an organic pollutant, and the spherical particles exhibited the highest rate constant, followed by penta/hexagonal, triangles, and nanosheets, respectively. In a follow up study, a similar process was used to generate various morphologies of AgNPs such as spherical (2–5 and 40–50 nm), rectangular, and penta/hexagonal particles. These AgNPs were used as an antibacterial agent against multidrug-resistant bacteria, and small-sized spherical (2–5 nm) particles demonstrated maximum zone of inhibition, followed by pentagonal and hexagonal particles. At similar size (40–50 nm), penta/hexagonal NPs were found to be more effective than spherical NPs for antimicrobial activity [133]. In another study, *Lactobacillus plantarum* TA4 biomass and cell-free supernatant were used to generate nanoflower-shaped (291 ± 98.1 nm) and irregular (191.8 ± 69.1 nm) ZnO NPs, respectively. At same concentration, despite the larger size, nanoflower-shaped ZnO NPs exhibited a higher cytotoxicity toward Vero cell line, with an IC_50_ value of 55 μg/mL, compared to 100 μg/mL of irregularly shaped particles [134]. Size and protein corona present on the surface of the AuNPs have been shown to affect the catalytic properties. Das et al. [135] studied one pot synthesis of AuNPs from protein extract of *R. oryzae* and produced AuNPs ranging from 5 to 65 nm in size, by changing the HAuCl_4_ to protein extract ratio. These AuNPs were used for the degradation of *p*-nitrophenol, and the authors reasoned that induction time and reaction rate is dependent on size of NP and protein corona. For smaller AuNPs, the thickness of the protein layer limited the diffusion, while for the larger particles the reduction in available surface area reduced the reaction rate. In a similar study, Qu et al. [136] used cell-free extracts of *Magnusiomyces ingens* LH-F1 and varied the concentration of extract from 25 to 200 mg/L to produce AuNPs ranging from 28.3 to 20.3 nm, with uniform size distribution at smaller size. The smaller particles exhibited lower apparent reaction rate and longer induction periods during catalytic degradation of 4-nitrophenol. The authors hypothesized that at high concentrations of extract, smaller particles with a thicker protein corona were formed, affecting the diffusion and adsorption. In contrast to the effect of active components present on the NP surface, Ag_2_O/AgO-TiO_2_ nanocomposites biosynthesized by *Alcaligenes aquatilis* used for photocatalytic degradation of Reactive Blue 220 showed that calcined nanocomposite exhibited slightly better degradation, indicating that biomolecules were not required for photocatalytic activity. The calcination at 450 °C for 3 h removed the organic functional groups and enhanced the crystallinity of the nanocomposite. However, the authors recommended the use of as-synthesized nanocomposites for photocatalysis instead of calcined one owing to the higher energy requirement and only a marginal improvement in degradation [137].

Kasemets et al. [138] studied the concerted effects of surface charge and size on *Saccharomyces cerevisiae* BY4741 cells using 10- and 80-nm AgNPs coated with either positively or negatively charged molecules. Exposure of yeast cells to these particles exerted an expected size-based effect, but positively charged NPs were 8–44 times more toxic than the negatively charged particles. Microscopic analysis showed that the positively charged particles were bound to the yeast cell surface. The positive charge on the surface of silica NPs has also been correlated with their better uptake by the 3T3-L1 cell line, although this result was found to be cell type dependent [139]. Another study investigated the effect of surface charge on the uptake of AuNPs in four plant species and found that positively charged particles were taken up by the roots, whereas negatively charged particles were transported into the plant shoots [140]. The negative zeta potential of cerium oxide NPs (CeO_2_NPs), along with their round shape and biocorona formation, has been shown to influence cytotoxicity in mussel hemolymph [141]. CeO_2_NPs have also been shown to exhibit pH-dependent antimicrobial action against *P. aeruginosa* and *S. epidermis*, which was attributed to the smaller size of the generated particles and the negative surface charge [142]. The surface charges on titania NPs have been shown to affect the photocatalytic degradation of methylene blue. The NPs prepared at pH 7 and 10 displayed negative charges on their surfaces and were able to adsorb cationic dye [143].

**Fig. 7:**
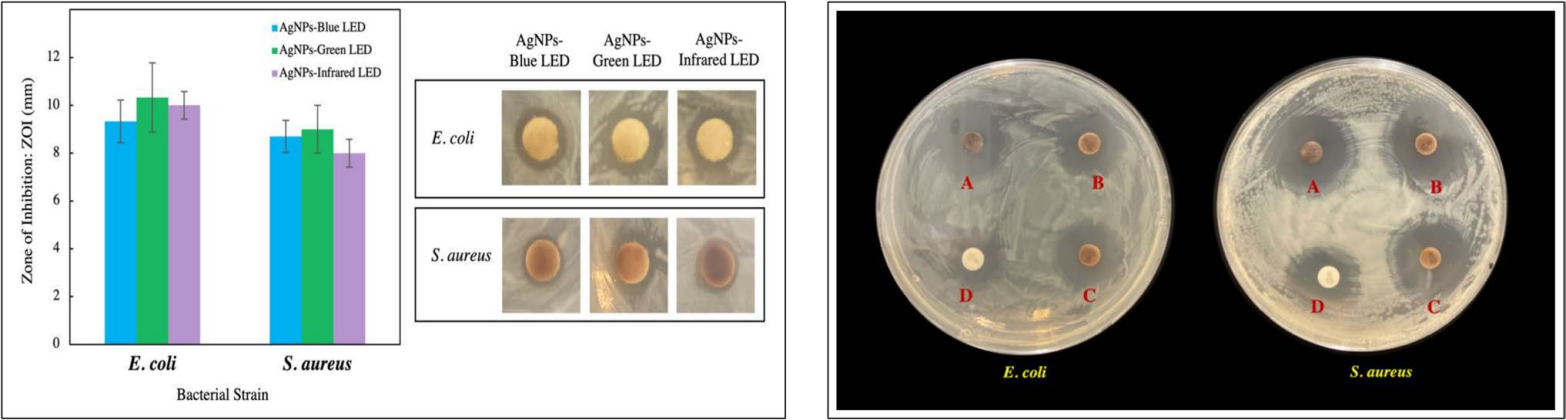
Antibacterial activity of light-emitting diode (LED)-mediated biosynthesized silver nanoparticles (AgNPs). Effect on *Escherichia coli* and *Staphylococcus aureus* (left panel). Symbiotic effect after conjugation with streptomycin (right panel), where the bacteria were exposed to (A) blue LED AgNPs-streptomycin, (B) green LED AgNPs-streptomycin, (C) infrared LED AgNPs-streptomycin, and (D) streptomycin only. Adapted from [121], MDPI (CC BY 4.0.)

The effect of light irradiation on AuNPs synthesized via sodium citrate reduction has been studied previously. Irradiation of a synthesized colloidal solution with different light wavelengths increased the absorption maxima and changed the position of the SPR band. Moreover, the absorption of green light changed the spherical shape of the NPs into a branched nano-urchin-type morphology. AuNPs irradiated with green light were found to increase the production of catalase and superoxide dismutase in *Penicillium chrysogenum* at 14 days, whereas malondialdehyde levels were found to be weakly correlated with increased light intensity [144]. In another study, AgNPs biosynthesized with LED exposure were shown to have antimicrobial efficacy that correlated with the surface charge. The AgNPs synthesized in the presence of green LEDs exhibited the highest surface charge and antimicrobial activity (calculated as the zone of inhibition), despite having a larger average particle size than that of the other treatments (Fig. 7). The study also investigated the effect of LED-treated AgNPs in conjunction with the standard antibiotic, streptomycin. The effect of combined treatment was also found to be highest for AgNPs synthesized in the presence of green LEDs, and all the AgNPs exhibited a fractional inhibitory index ≤ 0.5, indicating a synergistic effect [121]. Overall, the discussed literature clearly supports that the size, shape, and surface charge, among other factors, influence the properties and efficacy of NPs for various applications. Although some of the studies discussed in this section pertain to either chemically synthesized NPs or plant extract-mediated synthesis, they clearly elucidate the effects of physicochemical factors used during synthesis on the final properties of NPs. In that regard, these studies provide insights into the possible applications that can be accomplished once we are able to achieve the controllable synthesis of NPs via a microbial route.

## Challenges involved in biosynthesis and applications of nanoparticles

The current microbe-mediated synthesis processes have several limitations that must be overcome before touting microbial biosynthesis as the panacea to the other methods. A major drawback undoubtedly is the control of size, distribution, polydispersity, and shape of the synthesized NPs. This is compounded by the fact that different microorganisms have different growth requirements and use varied processes for NP generation. The extraction of the NPs is also challenging, especially for the intracellular synthesis, but even for the extracellular synthesis, simple techniques such as centrifugation might cause irreversible harm to the NP properties. Hence, the effect of upstream and downstream processing on the NP properties should also be studied. Additionally, scaling up of microbe-mediated NP synthesis remains unaccomplished as of now. Therefore, to understand and ensure optimal parameters for controlled NP synthesis, it is necessary to understand the mechanistic pathways involved and various physicochemical parameters that affect these pathways. Apart from microbial production, another challenge involved in the use of NPs for various applications is their impact on the environment. Some metallic NPs can have a detrimental effect on aquatic habitats or on the growth of plants [145]. Hence, it is also important to understand the transport behavior of the NPs.

## Conclusion and future perspectives

The microbiological route for the synthesis of NPs is an interesting alternative to physical and chemical methods. Given the enormous number of microorganisms present in nature, it is conceivable that microbe-mediated processes can be more flexible in terms of the type of NPs that can be synthesized and might be able to provide better control over this process. This review focused on the various possible mechanisms of NP synthesis employed by microorganisms, the current status and research in this sphere, the effects of numerous physicochemical parameters on the synthesis and applications of various NPs, and the future challenges and applications. Hopefully, in future, more efficient organisms can be isolated from microbial dark matter that can be harnessed for the biosynthesis of NPs. In addition, advances in understanding the mechanistic principles of NP generation could help create recombinant strains that can be used for efficient production. Already, recombinant strains have been created for the production of metallic NPs by utilizing the genes and mechanistic principles involved in microbial NP generation. Chen et al. [146] showed the production of CdSNP in recombinant *E. coli* by overexpression of glutathione synthetase, inspired from yeast CdSNP synthesis mechanism, with production exceeding the yield of NPs from yeasts. Other studies have utilized recombinant *E. coli* co-expressing a metal-binding protein, metallothionein, and phytochelatin synthase that synthesizes a metal-binding peptide phytochelatin to produce various nanomaterials, including some that were never previously synthesized [147, 148]. Another study was able to produce thermostable magnetic NPs by purifying a thermostable ferritin named PcFn, which was overexpressed in recombinant *E. coli* [149]. A *Pichia pastoris* strain overexpressing cytochrome b5 reductase has been used for production of AgNPs and selenium NPs [150]. These studies illustrate the potential of genetic engineering in microbial NP production; however, so far large-scale production remains elusive. The future acceptance of microbial synthesis as a valid alternative for current methods will largely depend upon the cost-efficiency of the process. Thus, the future of microorganisms as nano-factories can possibly only be achieved by gaining an overall understanding of the various facets of NP production with synergistic application of ever-increasing information and technology to ensure that microbial-based processes can compete economically with the physical and chemical processes typically used for NP synthesis.

## Data Availability

No datasets were generated or analysed during the current study.

## Declarations

### Data transparency

Not applicable/not available.

### Research data

Not applicable/not available.
